# A Novel Inverse Analysis Method for Mechanical Parameter Acquisition in SiC_f_/SiC Composites and Its Application to Turbine Disc Damage Assessment

**DOI:** 10.3390/ma18010160

**Published:** 2025-01-02

**Authors:** Wenjun Wang, Qi Zeng, Chaochao Li, Min Li, Liang Cao, Guoqing Chen, Peng Cao

**Affiliations:** 1 AECC Hunan Aviation Powerplant Research Institute, Zhuzhou 412002, China; mifeng_2005@163.com (W.W.); licc201903@163.com (C.L.); limin0093@163.com (M.L.); 2 School of Aerospace Engineering, Xiamen University, Xiamen 361102, China; 3 College of Architecture and Civil Engineering, Beijing University of Technology, Beijing 100124, China; caol@emails.bjut.edu.cn; 4 School of Architecture and Engineering, Northeast Electric Power University, Jilin 132012, China; 2202200935@neepu.edu.cn

**Keywords:** turbine disc, SiC_f_/SiC composites, parametric inverse analysis, finite element simulation, damage assessment

## Abstract

Obtaining the mechanical parameters of SiC_f_/SiC composites quickly and accurately is crucial for the performance evaluation and optimal design of novel turbine disc structures. A representative volume element (RVE) model of 2D woven SiC_f_/SiC composites was developed using CT scanning and machine learning-driven image reconstruction techniques. The stress-strain curve was obtained by uniaxial tensile test, and the anisotropic mechanical parameters were obtained by inverse analysis using a non-dominated sorting genetic algorithm (NSGA-II). Subsequently, the uniaxial tension simulation was carried out based on the RVE model and mechanical parameters. The results show that the simulation curve is in good agreement with the test, and the errors of initial modulus and peak stress were 3.98% and 2.75%, respectively. Finally, the finite element models of the turbine disc with two braiding schemes were established to simulate the damage of the turbine disc. And the simulation results were verified by a centrifugal test. The failure modes of the two kinds of turbine discs are similar to the centrifugal test results, and the maximum rotating speed was close to the test results. The findings of this study provide a novel solution for obtaining the anisotropic mechanical parameters of SiC_f_/SiC composites with different woven schemes.

## 1. Introduction

As the core component between the combustor and the nozzle, the performance of the turbine disc plays a crucial role in the thrust-weight ratio, thermal efficiency, and life of the aero-engine [1,2,3]. The traditional turbine disc made of alloy materials makes it challenging to meet the performance requirements of a high thrust-weight ratio aero-engine because of the limited space for weight loss and high-temperature performance improvement [4,5]. In response, the continuous fiber-reinforced ceramic matrix composite with high-temperature resistance, low density, high specific strength, oxidation resistance, ablation resistance, and high reliability has become the preferred material for turbine disc structure in aero-engines [6,7,8,9].

The continuous fiber-reinforced ceramic matrix composites can be divided into C/SiC [10] and SiC_f_/SiC [11]. Nevertheless, like all carbonaceous materials, carbon fibers exhibit poor high-temperature oxidation resistance [12,13]. Therefore, SiC_f_/SiC composites are the primary materials in turbine discs. However, SiC_f_/SiC composites differ from metallic materials in their stiffness and strength characteristics due to the influence of the braiding process, which is markedly anisotropic [14,15,16]. The damage behavior of SiC_f_/SiC composite structures is currently evaluated utilizing tensile [17,18,19], compression [20,21], and shear tests [22,23]. However, evaluating the anisotropic properties of SiC_f_/SiC composites through experimental tests alone is costly in terms of raw materials and time, and it also increases the iteration cycle and development cost of new turbine disc structures.

Numerical methods have been used to evaluate the properties of SiC_f_/SiC composites to solve these problems. Based on the periodic boundary condition, Chen and Liu [24] established the meso-element model of two-dimensional woven SiC_f_/SiC composites, and the macroscopic stress-strain response and progressive damage process of the material under uniaxial tensile, compressive, and in-plane shear loads were simulated. The results show that the finite element simulation results agree with the experimental results. Han et al. [25] conducted a multi-scale fatigue analysis of 2D woven SiC_f_/SiC composites using the general cell program and finite element simulation software. They found that the simulation results accurately predicted the fatigue life of the components. Wang et al. [26] developed a mesoscale finite element model incorporating yarn shapes and pore defects based on CT scan images of 3D hexagonally braided SiC_f_/SiC composites. The errors between the simulated modulus, stress and strain, and the uniaxial tensile test are 3%, 9%, and 5%, respectively. Yan et al. [27] reconstructed a macroscopic finite element model of a three-dimensional wound tube of SiC_f_/SiC composites containing microporous structural features by combining CT data and the principle of minimum potential energy and found that the simulation results were similar to those of circumferential compression.

All the above studies show that the strength characteristics and damage of SiC_f_/SiC composites can be evaluated well using the finite element simulation technique. Nevertheless, the model input parameters for the above studies were still obtained through a variety of mechanical (compression, tensile, and shear) tests. This also indirectly increases the time cost of the simulation and generates material consumption. Moreover, due to the anisotropy of the SiC_f_/SiC composites, it is difficult to obtain all the mechanical parameters only through experiments. In this regard, an inverse analysis method has been proposed by scholars [28,29] to quickly obtain some mechanical parameters of the materials and achieve better results. Therefore, an inverse analysis method is critical for obtaining mechanical parameters of SiC_f_/SiC composites in all directions rapidly and accurately based on the unidirectional test results. Furthermore, it can provide material parameter support for the performance evaluation and optimization of turbine discs under different braiding schemes.

**Figure 1:**
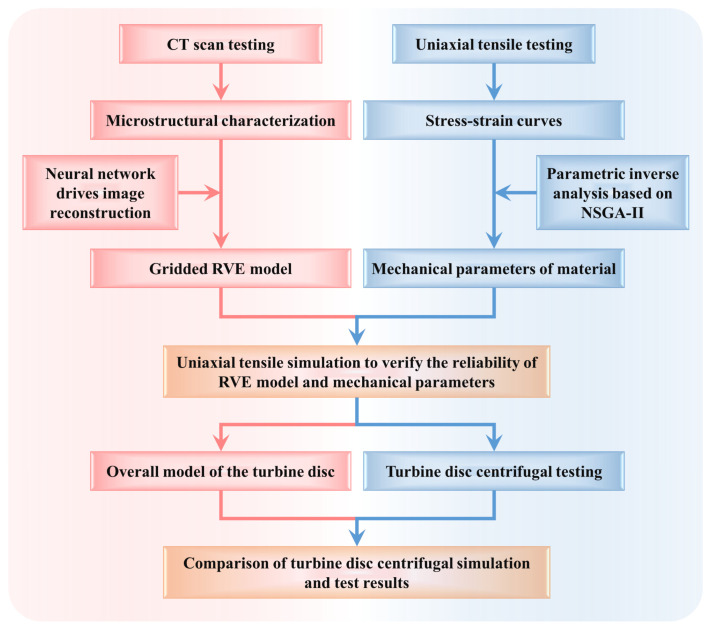
The research technology roadmap for this study.

Based on this, a fast and accurate inverse analysis method is proposed to obtain the mechanical properties of 2D woven SiC_f_/SiC composites, and the damage of the turbine disc under the two braiding schemes is evaluated using the finite element simulation technique. The technical route of this study is shown in Figure 1. Firstly, the meso-structural characteristics of SiC_f_/SiC composites are obtained based on CT scanning technology. And the RVE model with material structure features is reconstructed using a deep learning neural network in Dragonfly v2.1.0 software. Then, an inverse analysis of the uniaxial tensile stress-strain curves of the SiC_f_/SiC composites is carried out using NSGA-II to obtain the isotropic mechanical parameters. Subsequently, the obtained material parameters are used as input values of the RVE model for uniaxial tensile simulation. The uniaxial tensile simulation results are compared with the experimental results to verify the reliability of the RVE model and the inverse analysis method. Finally, based on the above parametric inverse analysis method, the damage of the turbine disc is simulated under different weaving schemes and rotating speeds and verified by a centrifugal test. The results of this study provide a new method for obtaining the anisotropic mechanical parameters of SiC_f_/SiC composites with different braiding schemes.

## 2. Specimens and Experiments

### 2.1. Raw Materials

**Figure 2:**
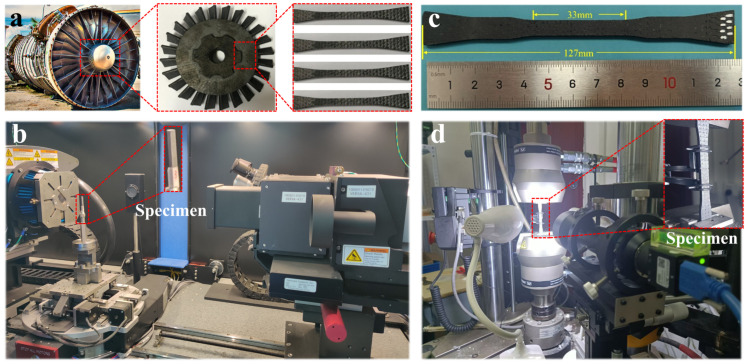
The raw materials and test equipment. (**a**) Physical drawing of turbine disc. (**b**) CT scan testing diagram. (**c**) Uniaxial tensile testing specimen size. (**d**) Uniaxial tensile testing diagram.

The 2D woven SiC_f_/SiC composites used in this experiment were the same as that of the turbine disc of an aero-engine, as shown in Figure 2a. The SiC_f_/SiC composites were composed of woven SiC fiber preform and SiC matrix. The longitudinal and transverse fiber bundle woven density were 8 bundles/cm and 4 bundles/cm, respectively. The corresponding plain weave density was 216 g/m^2^, and the fiber volume fraction was 33.7 vol%.

### 2.2. CT Scan Testing

To investigate the microstructure and porosity distribution of 2D woven SiC_f_/SiC composite, the samples were scanned by CT equipment. The porosity was determined according to the density of warp and weft and the scanning of the samples. As shown in Figure 2b, the model of the CT scanning equipment is nanoVoxel–3000 produced (Tianjin Sanying Precision Instruments Co., Ltd., Tianjing, China). The device has a pixel detail resolution of 500 nm, a spatial resolution of 2 μm, an imaging area of 427 mm × 427 mm, and a pixel matrix of 3072 × 3072. A 5 mm × 5 mm area in the X–Y plane was selected as the standard sample size, and the 3D visualization was carried out using professional image processing software AVIZO 2019.

### 2.3. Uniaxial Tensile Testing

The specimen size for uniaxial tensile testing is shown in Figure 2c, where the specimen was 3 mm thick. Three samples were taken for testing, and the average of the test results was compared with subsequent simulation results. The tensile test was carried out at room temperature by the Instron 8872 electro-hydraulic servo fatigue testing machine (Instron, Norwood, MA, USA). The test system is shown in Figure 2d. The friction at the specimen/clamping interface was used to transfer the tensile load imposed by the testing machine. According to the ASTM C1359 test standard [30], the tensile loading rate was set to 0.3 mm/min, which ensured quasi-static loading during the tensile process.

### 2.4. Centrifugal Testing

**Figure 3:**
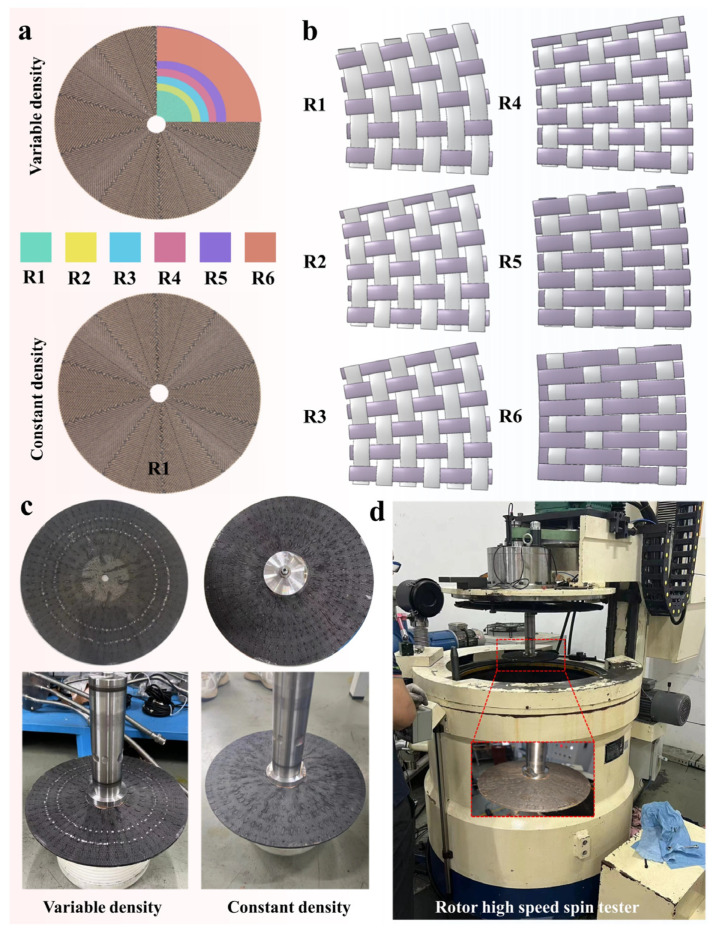
Schematic construction and centrifugal test diagrams of two turbine discs. (**a**) Schematic diagrams of two turbine discs. (**b**) Schematic illustration of different weaves in a variable density turbine disc. (**c**) Actual picture of two types of turbine discs. (**d**) Centrifugal testing diagram.

This study used the turbine disc structures of two fiber-based formulation schemes for centrifugal testing. As shown in Figure 3a, one is a variable density woven turbine disc, and the other is a constant density woven turbine disc. Specifically, the variable density design was determined by our preliminary simulations based on the stress distribution when the turbine disc is subjected to centrifugal loading. The variable density turbine disc was composed of six kinds of woven fabrics, and the fabric structure under different braiding methods is shown in Figure 3b. This design takes into account the different parts in the work of the mechanical performance requirements by adjusting the weaving density to optimize the overall structure. The warp and weft density values corresponding to the above six weaving schemes are shown in Table 1. It can be noticed that with the increase of the distribution radius, the longitudinal direction of the circular fabric is becoming denser, and the latitudinal direction is becoming less dense. The whole constant density turbine disc was woven like R1 in the variable density turbine disc. This scheme simplifies the manufacturing process even more, but in some cases, it may not be as targeted and adaptable as the variable density scheme above. The two turbine disc samples used for centrifuge testing are shown in Figure 3c. The average thickness of both samples was 5.5 mm, with an inner aperture of 12.2 mm and an outer diameter of 277.2 mm. Moreover, three turbine discs of each type were used for the centrifugal testing, and the average of the destructed rotational speeds was taken as a contrasting value for the subsequent simulation results. A high-speed rotating test bed (Hengchao Installation and Testing Technology Co., Ltd., Beijing, China) with the highest speed of 40,000 RMP was used to test the turbine disc, and the test instrument is shown in Figure 3d.

## 3. Development of the RVE Model and the Turbine Disc Model

### 3.1. RVE Model

**Figure 4:**
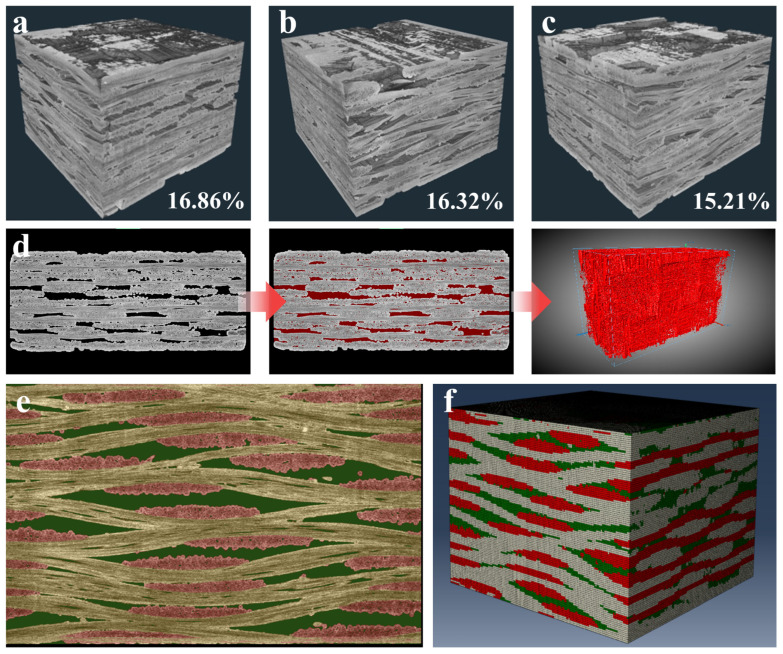
CT scanning results and RVE model building. (**a**) Upper position scanning results. (**b**) Central position scanning results. (**c**) Lower position scanning results. (**d**) Pore extraction process. (**e**) Segmentation results for each part. (**f**) RVE model.

The visualization model of 2D woven SiC_f_/SiC composites at three positions after treatment by AVIZO 2019 software is shown in Figure 4a–c. It can be seen that the SiC_f_/SiC composites are mainly composed of three parts, namely, SiC fiber bundles, SiC matrix, and pores. Moreover, it is found from Figure 4a–c that the SiC matrix fills into the fiber bundles more densely, but the surface of the whole fabric is not smooth. There are a lot of unfilled areas between the longitudinal and zonal fiber bundles, so porosity is an essential part of the composite at the meso-scale.

The pores were statistically analyzed using the Label Analysis function of the Avizo 2019 Software [31,32], and the results are shown in Figure 4d. The specific porosity is also shown in Figure 4a–c, and the pore volume content of the SiC_f_/SiC composites is 15–17%. Compared with other image processing software, the Dragonfly v2.1.0 software has been used by many scholars for segmentation processing of CT images because it contains a machine learning function [33,34,35]. Therefore, to obtain a finite element model for simulation analysis, this study first trained the model on multi-slice CT images in a 5-layer U-net architecture depth learning module in Dragonfly v2.1.0 software. Considering the directivity of x- and y-direction fibers in SiC_f_/SiC composites, it is difficult to distinguish them by grayscale. Therefore, the longitudinal fibers, zonal fibers, and matrix were segmented by manual adjustment in this study. Then, the segmented CT images were used as training objects for the segmentation model, and the training parameters and enhancement parameters were continuously adjusted. Subsequently, the trained model was utilized to process other CT images. Finally, the machine learning processed images were compared with the manually segmented comparison images. When the model is 95% correct, the model was considered complete. Finally, the CT images of the SiC_f_/SiC composites were segmented using the trained model in Dragonfly v2.1.0.

Figure 4e shows the segmented image of the SiC_f_/SiC composites. The three components of SiC_f_/SiC composites, namely, longitudinal fiber, zonal fiber, and matrix, are separated and identified. Subsequently, the segmented image files were transformed into mesh models using the Python 3.11 programme written in this study and imported into ABAQUS 2022. Finally, the RVE model of the SiC_f_/SiC composites can be obtained from CT images as shown in Figure 4f. Moreover, the dimensions of the RVE model are consistent with the CT images exactly. The radial fibers are shown in white, the zonal fibers in red, and the matrix in green. The model contains approximately 850,000 hexahedral units with a mesh size of 5 μm. In particular, this study employed the homogenization method to equate the pore portion of the SiC_f_/SiC composites within the matrix to reduce the difficulty of meshing.

### 3.2. Turbine Disc Model

**Figure 5:**
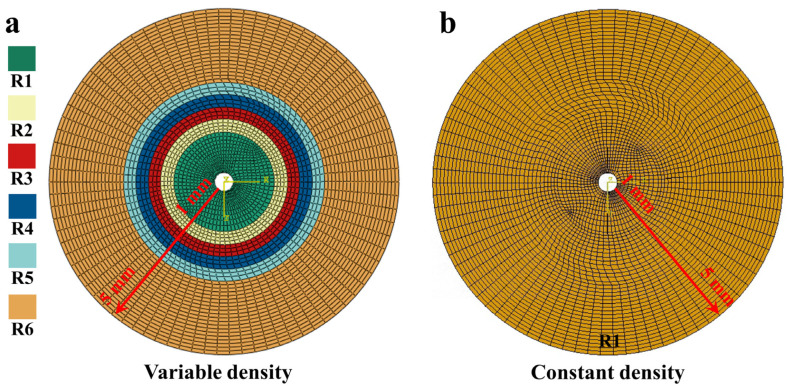
Two types of turbine disc models. (**a**) Constant density. (**b**) Variable density.

Figure 5 shows the mesh models of two types of turbine disc. The geometry of the two models is identical to that of the specimens. The number of meshes and nodes are 6990 and 10,692, respectively. The mesh type is a hexahedral element (C3D8R) with reduced integration. In order to improve the computational accuracy of the model and reduce the computational time, the variable-size mesh was used in this study. As shown in Figure 5, the mesh size of the model linearly increases from the inside to the outside along the radius. Of these, the mesh size at the model’s center is 1 mm, and the mesh size at the outer edge is 5 mm. Then, the radial displacement at the circular hole of the turbine model and the rotation displacement in the x, y and z direction is restrained, and centrifugal force is applied to the disc model to simulate the centrifugal testing process. Finally, the ABAQUS2021 Standard module was used to solve the model.

### 3.3. Uniaxial Tensile Test Simulation

There is no research to establish a more complete strength criterion for ceramic matrix composite fiber bundles [36,37]. In recent years, researchers [38,39,40] have tended to think of fiber bundles as transversely isotropic monolayer fiber-reinforced composites and have used strength theory for veneer composites to make predictions. The most widely applied strength criterion is the Hashin criterion, which considers different failure modes in fiber-reinforced composites and can characterize the damage profile of the composites at failure [41,42,43]. For this reason, this study used the three-dimensional Hashin criterion based on failure strain as the strength criterion of fiber bundles. The three-dimensional Hashin criterion classifies the failure of composites into fiber-dominated failure types and matrix-dominated failure types, specifically into four failure modes [44,45].

(1) Fiber-dominated tensile failure (*σ*_1_ ≥ 0):

(1) $$F_{ft}={\left(\frac{\sigma_{11}}{X_{T}}\right)}^{2}+\alpha {\left(\frac{\sigma_{12}}{S_{12}}\right)}^{2}+{\left(\frac{\sigma_{13}}{S_{13}}\right)}^{2}\geq 1$$

(2) Fiber-dominated compression failure (*σ*_1_ < 0):

(2) $$F_{fc}={\left(\frac{\sigma_{11}}{X_{C}}\right)}^{2}\geq 1$$

(3) Matrix-dominated tensile failure (*σ*_2_ + *σ*_3_ ≥ 0):

(3) $$F_{mt}={\left(\frac{\sigma_{22}+\sigma_{33}}{Y_{T}}\right)}^{2}+\frac{\sigma_{23}^{2}-\sigma_{22}\sigma_{33}}{S_{23}^{2}}+{\left(\frac{\sigma_{12}}{S_{12}}\right)}^{2}+{\left(\frac{\sigma_{13}}{S_{13}}\right)}^{2}\geq 1$$

(4) Matrix-dominated compression failure (*σ*_2_ + *σ*_3_ < 0):

(4) $$F_{mc}={\left(\frac{\sigma_{22}+\sigma_{33}}{2S_{23}}\right)}^{2}+\frac{\sigma_{22}+\sigma_{33}}{Y_{C}}\left[{\left(\frac{Y_{C}}{2S_{23}}\right)}^{2}-1\right]+\frac{\sigma_{23}^{2}-\sigma_{22}\sigma_{33}}{S_{23}^{2}}+{\left(\frac{\sigma_{12}}{S_{12}}\right)}^{2}+{\left(\frac{\sigma_{13}}{S_{13}}\right)}^{2}\geq 1$$

where *σ_ij_* (*i*, *j* = 1, 2, 3) are the principal stress tensor values, *X_T_*, *X_C_*, *Y_T_* and *Y_C_* are the tensile and compressive strengths in the fibre and matrix directions respectively. The values *S*_12_*, S*_13_ and *S*_23_ represent the shear strengths in different directions and α is a parameter which controls the influence of the shear stress in tensile failure.

Due to the addition of randomly distributed pore units in the matrix, these pore units have been approximately representative of the matrix tensile strength heterogeneity. Therefore, the SiC matrix unit in this study no longer obeys the random Weibull tensile strength distribution but uses the unified SiC matrix tensile strength value.

### 3.4. Centrifugal Test Simulation

The Tsai–Wu failure criterion [46,47] is a phenomenological failure theory widely used in anisotropic composites with different tensile and compressive strengths. The Tsai–Wu criterion predicts a failure state when the failure index of the material reaches 1. The failure criterion is a particular case of the general secondary failure criterion and can be expressed in the following form:

(5) $$F_{i}\sigma_{i}+F_{ij}\sigma_{i}\sigma_{j}\leq 1$$

Among them, *F_i_* and *F_i__j_* are intensity parameters obtained from experiments, *σ_i_* and *σ_j_* are marked by Voigt with second-order tensor, and the interaction term *F_ij_* also needs to satisfy the following constraints:

(6) $$F_{ii}F_{jj}-F_{ij}^{2}\geq 0$$

This means that the *F_ij_* term must be positive. For orthotropic materials with three symmetric planes, if *F_ij_* = *F_ji_*, and if there is no coupling between everyday stress and shear stress, and between shear stress and shear stress, the general form of Tsai–Wu failure criterion is simplified as follows:

(7) $$\begin{array}{c} F_{1}\sigma_{1}+F_{2}\sigma_{2}+F_{3}\sigma_{3}+F_{4}\sigma_{4}+F_{5}\sigma_{5}+F_{6}\sigma_{6}\\ +F_{11}\sigma_{1}^{2}+F_{22}\sigma_{2}^{2}+F_{33}\sigma_{3}^{2}+F_{44}\sigma_{4}^{2}+F_{55}\sigma_{5}^{2}+F_{66}\sigma_{6}^{2}\\ +2F_{12}\sigma_{1}\sigma_{2}+2F_{13}\sigma_{1}\sigma_{3}+2F_{23}\sigma_{2}\sigma_{3}\leq 1 \end{array}$$

In general, the uniaxial tensile and compressive strength of orthotropic materials in three directions are expressed as *σ*_1t_, *σ*_1c_, *σ*_2t_, *σ*_2c_, *σ*_3t_, and *σ*_3c_, and the shear strength is expressed as *S*_12_, *S*_23_, and *S*_31_. Then, the coefficients of the failure criterion for the orthotropic Tsai–Wu criterion are:

(8) $$\begin{array}{l} F_{1}=\frac{1}{\sigma_{1t}}-\frac{1}{\sigma_{1c}};\;F_{2}=\frac{1}{\sigma_{2t}}-\frac{1}{\sigma_{2c}};\;F_{3}=\frac{1}{\sigma_{3t}}-\frac{1}{\sigma_{3c}};F_{4}=F_{5}=F_{6}=0\\ F_{11}=\frac{1}{\sigma_{1c}\sigma_{1t}};\;F_{22}=\frac{1}{\sigma_{2c}\sigma_{2t}};\;F_{33}=\frac{1}{\sigma_{3c}\sigma_{3t}};\;F_{44}=\frac{1}{S_{23}^{2}};\;F_{55}=\frac{1}{S_{31}^{2}};\;F_{66}=\frac{1}{S_{12}^{2}} \end{array}$$

In the formula above, simple uniaxial tensile or shear tests can obtain *S*_12_, *S*_23_, and *S*_31_. In addition, *F*_1_, *F*_2_, *F*_3_, *F*_11_, *F*_22_, *F*_33_, *F*_44_, *F*_55_ and *F*_66_ can be expressed as:

(9) $$\begin{array}{l} F_{1}=\frac{1}{\sigma_{1t}}-\frac{1}{\sigma_{1c}};\;F_{2}=\frac{1}{\sigma_{2t}}-\frac{1}{\sigma_{2c}};\;F_{3}=\frac{1}{\sigma_{3t}}-\frac{1}{\sigma_{3c}};F_{4}=F_{5}=F_{6}=0\\ F_{11}=\frac{1}{\sigma_{1c}\sigma_{1t}};\;F_{22}=\frac{1}{\sigma_{2c}\sigma_{2t}};\;F_{33}=\frac{1}{\sigma_{3c}\sigma_{3t}};\;F_{44}=\frac{1}{S_{23}^{2}};\;F_{55}=\frac{1}{S_{31}^{2}};\;F_{66}=\frac{1}{S_{12}^{2}} \end{array}$$

There is a minus sign difference between the two, depending on whether the compressive stress is damaging in itself or if it is damaging in itself (negative); use the latter; otherwise, use the former. Theoretically, the coefficients *F*_12_, *F*_23_, and *F*_13_ can be determined by equal biaxial tests with the same stress in both directions. If the failure strength is equal to:

(10) $$\sigma_{1}=\sigma_{2}=\sigma_{b12},\;\sigma_{1}=\sigma_{3}=\sigma_{b13},\;\sigma_{2}=\sigma_{3}=\sigma_{b23}$$

*F*_12_, *F*_23_, and *F*_13_ can be expressed as:

(11) $$\begin{array}{c} F_{12}=\frac{1}{2\sigma_{b12}^{2}}[1-\sigma_{b12}(F_{1}+F_{2})-\sigma_{b12}^{2}(F_{11}+F_{22})]\\ F_{13}=\frac{1}{2\sigma_{b13}^{2}}[1-\sigma_{b13}(F_{1}+F_{3})-\sigma_{b13}^{2}(F_{11}+F_{33})]\\ F_{23}=\frac{1}{2\sigma_{b23}^{2}}[1-\sigma_{b23}(F_{2}+F_{3})-\sigma_{b23}^{2}(F_{22}+F_{33})] \end{array}$$

## 4. Parametric Inverse Analysis Based on NSGA-II Algorithm

After obtaining the uniaxial tensile stress-strain curves of the SiC_f_/SiC composites, the back analysis was performed by NSGA-II in Isight 2022 software to obtain the anisotropic mechanical parameters.

### 4.1. Basic Theory of NSGA-II

NSGA-II is an improved version of NSGA with strong exploration performance when dealing with highly nonlinear problems [48]. The most significant advantage of NSGA-II is that individuals close to the Pareto frontier are first selected in a non-dominant order, thereby improving Pareto’s ability to advance [49]. In addition, NSGA-II has the characteristics of good distribution and fast convergence speed and can get many high-quality solutions in a single run. In NSGA-II, a Simulated Binary crossover (SBX) approach is used as an operational mechanism for crossover and mutation [50] as follows:

Cross-operation:

(12) $$x_{i}^{\left(1,t+1\right)}=\frac{1+\beta_{qi}}{2}x_{i}^{\left(1,t\right)}+\frac{1-\beta_{qi}}{2}x_{i}^{\left(2,t\right)}$$

(13) $$x_{i}^{\left(2,t+1\right)}=\frac{1-\beta_{qi}}{2}x_{i}^{\left(1,t\right)}+\frac{1+\beta_{qi}}{2}x_{i}^{\left(2,t\right)}$$

Sudden mutation operation:

(14) $$x_{i}^{\left(1,t+1\right)}=x_{i}^{\left(1,t\right)}+\delta_{q}\left(x_{i}^{UB}-x_{i}^{LB}\right)$$

(15) $$\delta_{q}=\left\{\begin{array}{c} {\left[2u+(1-2u){\left(1-\delta \right)}^{\eta_{m}+1}\right]}_{\eta_{m}+1}^{1}-1,\;u\leq 0.5\\ 1-{\left[2(1-u)+2(u-0.5){\left(1-\delta \right)}^{\eta_{m}+1}\right]}_{\eta_{m}+1}^{1},\;u>0.5 \end{array}\right.$$

(16) $$\delta =\min\left(x_{i}-x_{i}^{LB},\;x_{i}^{UB}-x_{i}\right)/\left(x_{i}^{UB}-x_{i}^{LB}\right),u\in [1,0]$$

### 4.2. Parametric Inverse Analysis Process

**Figure 6:**
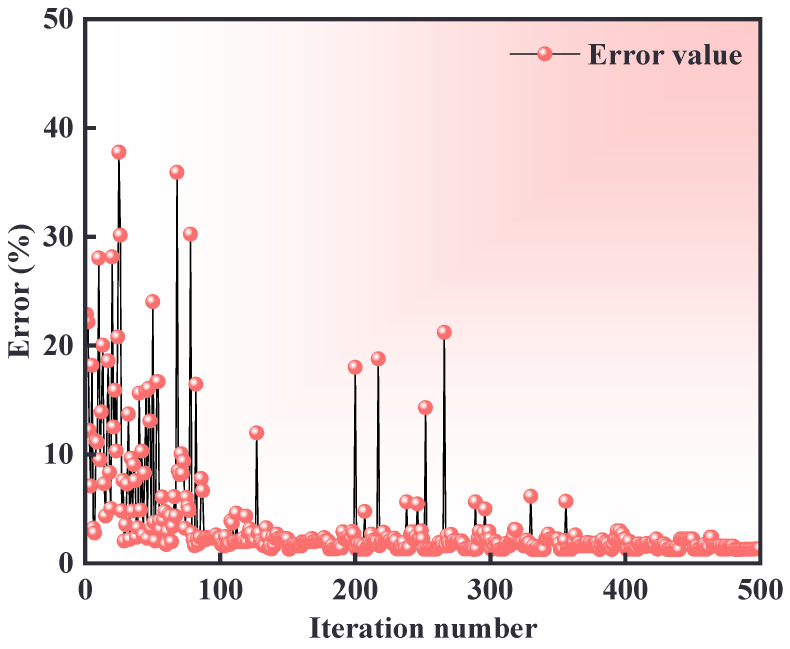
Iterative convergence process for the relative error.

The stress-strain curves obtained by uniaxial tension are imported into Insight 2022 software, and the optimization algorithm is selected as NSGA-II. Then, 40 iterative steps are set with precision controlled at 1 × 10^−6^. The initial values of Umat parameters, including the tensile, compressive, shear strength, and anisotropic modulus of the fiber are set, and the optimization interval is set. The minimum sum of squares of the relative error between the calculated and experimental results is taken as the objective function for the parameters inverse analysis, as shown in Equation (17). The convergence process and the relative error are shown in Figure 6.

(17) $$F=\sum_{i=1}^{n}{\left\{\frac{Q_{T}(i)-Q_{C}(i)}{\max(|Q_{T}(i)|,|Q_{C}(i)|)}\right\}}^{2}$$

where *Q*_T_(i) is the result of the trial; *Q*_C_(i) is the corresponding calculated result, and $\max(|Q_{T}(i)|,|Q_{C}(i)|)\neq 0$.

Finally, after fitting the experimental curves using the Insight 2022 software, the anisotropic mechanical parameters of the SiC_f_/SiC composites are obtained. The results are presented in Table 2 and Table 3.

## 5. Results and Discussion

### 5.1. Validation of RVE Model and Material Mechanical Parameters

**Figure 7:**
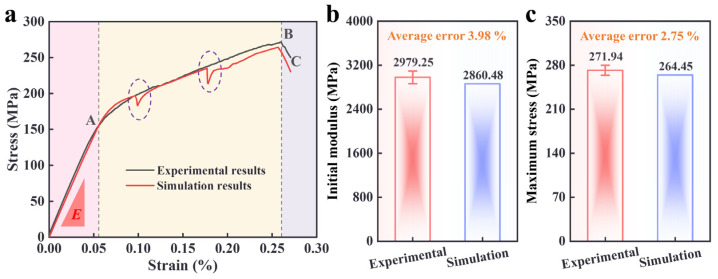
Comparison of uniaxial tensile test and simulation results. (**a**) Stress-strain curve. (**b**) Initial modulus. (**c**) Maximum stress. (A, B, C are the damage states of the specimens at different stages respectively. *E* is the initial modulus).

Taking the above mechanical parameters as the input values of the RVE model, the stress-strain curves of the SiC_f_/SiC composites were obtained by uniaxial tension simulation, as shown in Figure 7a. From Figure 7a, it can be found that the stress-strain curves of the finite element calculation results are in good agreement with the experimental results in each stage. Furthermore, two descending segments can also be noticed in the stress-strain curves obtained from the finite element simulation. Moreover, two descending segments can be observed in the simulated curves, which are mainly due to local fiber fracture. This also indicates that the finite element simulation can make up for the lack of real-time acquisition of experimental test data, thereby reproducing the real failure behavior of SiC_f_/SiC composites.

This study also compared the initial modulus and ultimate stress obtained by experiment and simulation, presented in Figure 7b and Figure 7c, respectively. From Figure 7b, it can be seen that the initial moduli obtained by experiment and simulation are 2979.25 MPa and 2860.48 MPa, respectively, and the error between them is only 3.98%. This shows that the linear elastic behavior of the material can be well reproduced based on the finite element simulation. It can be noticed from Figure 7c that the ultimate stress obtained by experiment and simulation is 271.94 MPa and 264.45 MPa, respectively, and the error between them is also tiny: only 2.75%. The results show that the mechanical parameters obtained from the RVE model and back analysis can simulate the in-plane uniaxial tensile mechanical response of a two-dimensional braided SiC_f_/SiC ceramic matrix composite.

**Figure 8:**
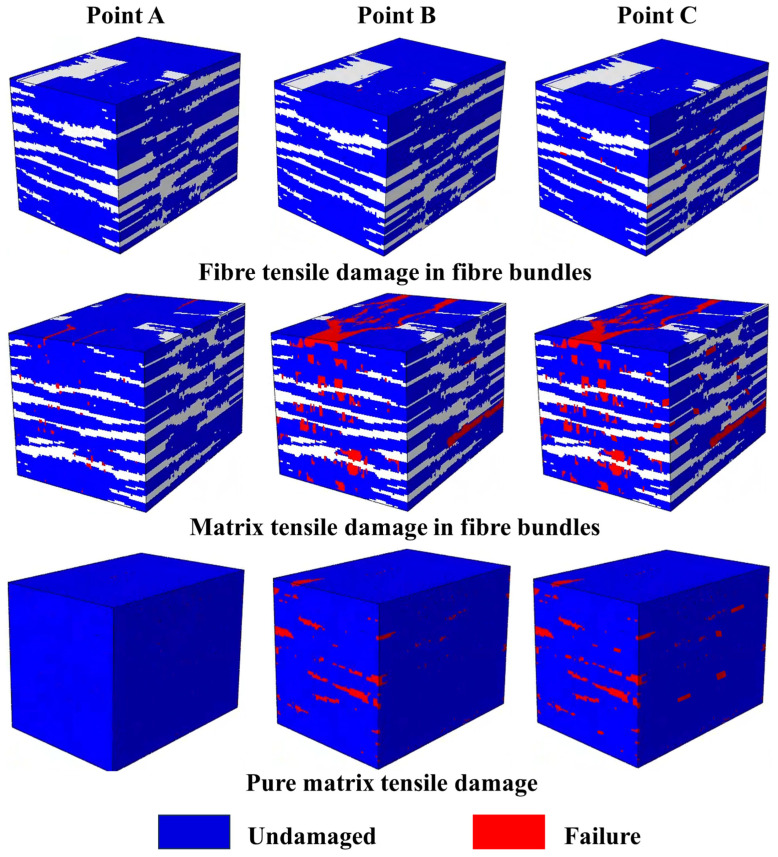
Damage of each component in the RVE model at different tensile stages. (The white section is the fibre).

Figure 8 shows the fiber-dominated tensile damage distribution in the fiber bundle, the matrix-dominated tensile damage distribution in the fiber bundle, and the pure matrix damage distribution for the RVE model at points A, B, and C in the stress-strain curve. Among them, the red part represents the damage to the fiber bundle unit or the matrix unit, and the blue part represents the undamaged matrix or the fiber bundle unit. When the strain is 0.06%, at point A, there is no damage to the matrix in the mesoscale cell. There is a small amount of matrix damage in the braided yarns perpendicular to the tensile direction, so the matrix damage in the fiber bundle perpendicular to the tensile direction is the main reason for the stiffness reduction of the AB segment. At point B, we can see that there is still no tensile damage in the bundle. However, the tensile damage of the matrix is much more than that of point A, and the matrix units begin to be damaged. From the pure matrix damage diagram, one can find the site where the matrix damage first appears. The damage is often in the periphery of multiple pores and the weakest place of the matrix unit in the single cell. The stress concentration caused by the pores is the leading cause of these lesions. The decrease of matrix content in the cross-section is also one of the reasons for the first matrix damage, and the matrix crack growth is perpendicular to the tensile direction. Point C represents the final failure stage of the RVE model when tensile damage begins to occur in the bundle. The fiber damage in the bundle often occurs from the edge of the yarn parallel to the drawing direction and gradually extends to the center of the yarn.

### 5.2. Comparison of Centrifugal Test and Simulation Results

**Figure 9:**
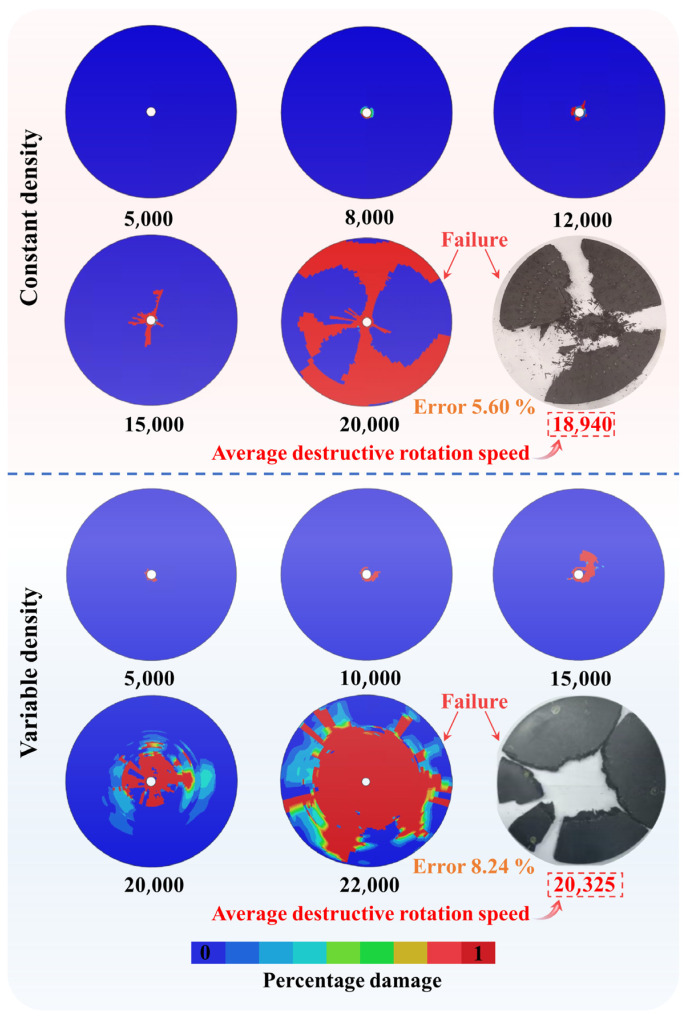
Turbine disc centrifugal test and simulation results.

The previous section verified that the mechanical parameters obtained by parameter inverse analysis are reliable in specimen-level samples. This section will further verify the reliability of the parameters from the turbine disc structure of the two braiding schemes. The simulation results of the two kinds of turbine disc structures at different rotating speeds and the final failure results are presented in Figure 9. It can be seen from Figure 9 that the constant density turbine disc is destroyed at a rotating speed of 18,940 r/min, and the destruction pattern is fragmentation into three fan-shaped parts. From Figure 9, it can be seen that when the rotating speed of the disc is 20,325 r/min, the shape of the variable density turbine disc is wholly broken up in the center and broken into several pieces at the edge. It also shows that the variable density design can be reasonably used to improve the ultimate speed of the turbine disc. However, considering the complexity of the manufacturing process of variable density turbine discs, performance and cost should be taken into account in practical applications.

The simulation results of two kinds of turbine discs at different rotating speeds are also presented in Figure 9. It can be noticed that when the rotating speed of the constant density turbine disc reaches 12,000 r/min, cracks appear at the center of the disc body first. Then, when the rotating speed reaches 20,000 r/min, the outer edge of the constant density turbine disc body breaks up, and the central clamping part gradually disintegrates and disintegrates. At 12,000 r/min, cracks first appeared in the center of the variable density disc. Subsequently, when the rotating speed reaches 22,000 r/min, the central part of the disc will be destroyed directly because of the vibration of the disc. The results of constant and variable density simulation show that the simulation pieces are damaged from the center of the disc body, and the fracture mode is split into fan-shaped fragments. The crack propagates directly along the radial direction until the crack develops to the outer edge of the disc body. However, a larger area of damage is found in the center of the variable density turbine disc, which is caused by the weak strength of the braided body.

In addition, by comparing the experimental results with the simulated ones, it can be seen that the error of the ultimate speed of the constant density turbine disc is 5.60%. In comparison, the variable density of the turbine disc is 8.24%. Moreover, the debris shapes of the two kinds of discs after destruction are similar to the simulation results. This shows that the material parameters obtained from the inverse analysis can be applied to the mechanical analysis of the turbine disc structure. Based on the simulation results, the meso-damage process of the turbine disc can be reproduced to optimize the structure of the turbine disc in time.

## 6. Conclusions

This study presents a fast and accurate inverse analysis method for obtaining the mechanical properties of two-dimensional woven SiC_f_/SiC composites, which is used to evaluate the damage of turbine discs under centrifugal load. The main findings are as follows:

1. The processing of CT scan images based on DragonFly can efficiently segment the components in SiC_f_/SiC composites, thus enabling the rapid construction of the RVE model with mesh.
2. The mechanical parameters obtained based on inverse analysis can well simulate the uniaxial tensile behavior of SiC_f_/SiC composites, wherein, the average errors of initial modulus and peak stress are 3.98% and 2.75%, respectively. Moreover, the simulated stress-strain curve can reproduce the local fiber fracture behavior that the experiment cannot observe.
3. The simulated damage location of the turbine discs under the two woven schemes is highly similar to the centrifugal test results. The maximum rotating speed of the damaged disc is close to the experimental results, and the errors of constant density and variable density disc are 5.60% and 8.24%, respectively.

The inverse analysis method proposed in this study can quickly and accurately obtain the mechanical property parameters of SiC_f_/SiC composites. This is of great significance in reducing the iterative cycles for the structural design of novel turbine discs. However, the SiC_f_/SiC composites used in this study are relatively monolithic, and the inverse analysis method needs to be improved in subsequent studies to allow it to be applied to various material configurations. Furthermore, the mechanical tests and parametric inverse analyses of SiC_f_/SiC composites in this study were performed only at room temperature. An attempt can be made in subsequent studies to establish an inverse analysis method capable of obtaining the mechanical parameters of SiC_f_/SiC composites in the high temperature environment.

## Figures and Tables

**Table 1:** Warp and weft densities of fabrics with different weaving patterns.

Distribution Area (Different Radius Ranges)	Warp Density (Root/cm)	Weft Density (Root/cm)	Fiber Volume Content (%)
R1 < 40	5	6	36.28
40 < R2 < 50	5.5	5.7	38.45
50 < R3 < 60	5.7	5.5	39.41
60 < R4 < 70	6.5	5.2	43.00
70 < R5 < 80	7	5	44.58
80 < R6 < 140	8	4	29.36

**Table 2:** Strength and stiffness parameters of fiber bundles.

Type	Symbol	Value
Axial tension strength	*X*_T_	1030 MPa
Axial compression strength	X_C_	1350 MPa
Transverse tension strength	Y_T_	59 MPa
Transverse compression strength	Y_C_	242 MPa
In-plane shear strength	S_12_	58 MPa
Out-of-plane shear strength	S_23_	60 MPa
Longitudinal elastic modulus	E_f_	390 GPa
Transverse elastic modulus	E_t_	72.4 GPa
Transverse shear modulus	S	4 GPa
In-plane Poisson’s ratio	V_12_	0.25
Transverse Poisson’s ratio	V_13_	0.45

**Table 3:** Stiffness parameters of matrix.

Type	Symbol	Value
Elastic modulus	E_m_	370 GPa
Poisson’s ratio	v_m_	0.35

## Data Availability

The original contributions presented in this study are included in the article. Further inquiries can be directed to the corresponding author(s).
